# Alleviating effect of Nexrutine on mucosal inflammation in mice with ulcerative colitis: Involvement of the RELA suppression

**DOI:** 10.1002/iid3.1147

**Published:** 2024-01-19

**Authors:** Hongyun Xu, Chunyu Wu, Danning Wang, Haiqiang Wang

**Affiliations:** ^1^ Graduate School Heilongjiang University of Chinese Medicine Harbin Heilongjiang China; ^2^ Department of Continuing Education First Affiliated Hospital of Heilongjiang University of Chinese Medicine Harbin Heilongjiang China; ^3^ Department of Liver, Spleen and Stomach First Affiliated Hospital of Heilongjiang University of Chinese Medicine Harbin Heilongjiang China

**Keywords:** inflammation, mucosal barrier damage, Nexrutine, RELA, ulcerative colitis

## Abstract

**Background:**

Nexrutine is an herbal extract derived from *Phellodendron amurense*, known for its anti‐inflammatory, antidiarrheal, and hemostatic properties. However, its effect on ulcerative colitis (UC) remains unclear.

**Methods:**

A mouse model of UC was induced by 3% dextran sulfate sodium, while human colonic epithelial cells NCM‐460 were exposed to lipopolysaccharide. Both models were treated with Nexrutine at 300 or 600 mg/kg, with Mesalazine applied as a positive control regimen. The disease activity index (DAI) of mice was calculated, and the pathological injury scores were assessed through hematoxylin and eosin staining. The viability of NCM‐460 cells was determined using the CCK‐8 method. Inflammatory cytokines were detected using ELISA kits. Expression of mucin 3 (MUC3), Claudin‐1, and tight junction protein (ZO‐1) was detected to analyze mucosal barrier integrity. Target genes of Nexrutine were predicted using bioinformatics tools. Expression of RELA proto‐oncogene (RELA) was analyzed using qPCR and western blot assays.

**Results:**

The Nexrutine treatments significantly alleviated DAI of mice, mitigated pathological changes in their colon tissues, decreased the production of pro‐inflammatory cytokines, enhanced the barrier integrity‐related proteins, and increased NCM‐460 cell viability in vitro. RELA, identified as a target gene of Nexrutine, showed elevated levels in UC models but was substantially suppressed by Nexrutine treatment. Adenovirus‐mediated RELA upregulation in mice or the overexpression plasmid of RELA in cells counteracted the effects of Nexrutine treatments, exacerbating UC‐related symptoms.

**Conclusion:**

This study demonstrates that Nexrutine alleviates inflammatory mucosal barrier damage in UC by suppressing RELA transcription.

## Graphical abstract

This preclinical research validates that Nexrutine indeed plays protective role against inflammatory mucosal injury in UC, which is due at least in part to the transcriptional suppression of RELA.

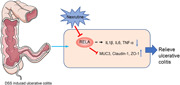

## 1 INTRODUCTION

Ulcerative colitis (UC) is a prominent form of inflammatory bowel disease, characterized by chronic, idiopathic inflammation of the colonic mucosa that can lead to disability.^1^, ^2^ Typically, this inflammation is confined to the mucosal surface, with the disorder often commencing in the rectum and progressively extending continuously to part of or the entire colon.^3^, ^4^ Clinical manifestations encompass recurring gastrointestinal tract inflammation, severe abdominal pain, diarrhea, and the presence of blood in stools.^5^ The global prevalence and morbidity of UC have been on the rise over time.^6^, ^7^ The primary pathogenic mechanism for UC involves aberrant activation immune responses, which are important defense guard against pathogenic factors but also culprit of disease progression.^8^ Mesalazine stands for the current standard regimen in the remission of mild to moderate disease; however, it necessitates up to 15 years of continuous treatment.^9^ Other biological agents, such as antitumor necrosis factor‐α (TNF‐α), anti‐integrin and anti‐interleukin (IL) agents, and the nonbiologic small molecule tofacitinib, can be administered in cases where there is an insufficient response to Mesalazine treatment.^10^ Nevertheless, the overall efficacy was limited by high recurrence rate and specific adverse effects.^11^ Consequently, ongoing basic and pharmacological investigations aim to develop innovative anticolitis drugs to address these unmet needs.

Nexrutine is a commercially available herbal extract from *Phellodendron amurense*, a widely used traditional Chinese medicine for the treatment of inflammation, abdominal pain, gastroenteritis, and diarrhea.^12^, ^13^ The *Phellodendron* plant, native to Asia, reportedly contains alkaloids, isoquinoline, flavone glycosides, and phenolic compounds.^14^ Subsequent studies have also highlighted the role of Nexrutine in cancer suppression.^15^, ^16^ Considering its anti‐inflammatory, antidiarrheal, and hemostatic properties, Nexrutine emerges as a potential candidate for the management of UC.

The nuclear factor‐κB (NF‐κB) transcription factor family plays a pivotal role in innate and adaptive immune responses and serves as a central orchestrator of the inflammatory process.^17^, ^18^ In mammals, the NF‐κB family comprises five transcription factors: c‐Rel, RelA (p65), RelB, NF‐κB1 (p105/p50), NF‐κB2 (p100/p52).^19^ Among them, RELA/p65, encoded by RELA proto‐oncogene, is a crucial member of the canonical NF‐κB pathway participates in inflammation modulation.^20^, ^21^, ^22^ Notably, the activation of RELA is closely associated with the development of UC.^22^ In this study, we identified the RELA gene as a candidate target of Nexrutine through bioinformatics. Consequently, we hypothesize that Nexrutine might exert specific alleviating effects on UC through the regulation of RELA.

## 2 MATERIALS AND METHODS

### 2.1 A mouse model of UC

Fifty‐six male C57BL/6J mice (6–8 weeks old, weighing 20–22 g) were procured from SJA Laboratory Animal Co., Ltd. and were housed in clean animal rooms at constant 22 ± 2°C, with free access to food and a lighting schedule of 8:00‐20:00. The use of animals was approved by the Animal Ethics Committee of First Affiliated Hospital of Heilongjiang University of Chinese Medicine (Approval number: HZYLLKY202201008). All procedures involving animal experiments adhered to the ARRIVE guidelines 2.0: Updated guidelines for reporting animal research.^23^ To induce UC, the mice were given drinking water containing 3% dextran sulfate sodium (DSS) for 7 days.^24^

Nexrutine (E3306; Selleck Chemicals) was administered to mice at two distinct doses (300 and 600 mg/kg), which were determined to be pharmacologically effective and safe based on a previous study.^25^ Mesalazine (S1681; Selleck) served as a positive control regimen. The daily dose of Mesalazine for clinical maintenance therapy in adults with UC is approximately 1.5 g. The mouse equivalent dose, adjusted based on body surface area between humans and mice, is calculated as follows: mouse equivalent dose = 1500 mg × 0.0026/0.02 kg = 195 mg/kg. The animals were assigned individual identification numbers. Subsequently, they were grouped into seven categories using a random number table, with eight mice in each group: Control group (given normal drinking water), DSS group (given 3% DSS), DSS + 300 mg/kg Nexrutine group (given 300 mg/kg Nexrutine and 3% DSS), DSS + 600 mg/kg Nexrutine group (given 600 mg/kg Nexrutine and 3% DSS), DSS + Mesalazine group (given 195 mg/kg Mesalazine and 3% DSS), DSS + Nexrutine + Ad‐NC group (given 300 mg/kg Nexrutine and 3% DSS, and injected with 100 µL Ad‐NC), DSS + Nexrutine + Ad‐RELA group (given 300 mg/kg Nexrutine and 3% DSS, and injected with 100 µL Ad‐RELA). Animal feed containing Nexrutine was administered from Days 1 to 12, and drinking water containing 3% DSS was given from Days 6 to 12. Mesalazine was administered from Days 1 to 12 by gavage. Adenovirus negative control (Ad‐NC) or Ad‐RELA was injected via the mesenteric artery on Day 6. In short, the mice were anesthetized by inhaled isoflurane and punctured through 1–2 cm of skin along the axillary line and the upper left side of the splenic region of the lateral abdomen. The external oblique abdominal muscle was incised to expose and separate the mesenteric artery, and the vascular abdominal aorta was clamped using a microvascular clamp. The injection needle was inserted into the mesenteric artery (predisposing colon infection) for adenovirus injection. The adenovirus was procured from HANBIO Technologies Co., Ltd. and administered at 100 μL (5 × 10^10^ vg) for each mouse.

After the 7‐day 3% DSS administration, the mice were euthanized by intraperitoneal injection of excessive (150 mg/kg) pentobarbital sodium, and their colon tissues were collected for subsequent analyses.

### 2.2 Disease activity index (DAI) evaluation

The body weight, stool consistency, and occult blood in stools of each mouse were recorded by a pathologist unaware of the mouse grouping details. Utilizing these data, the final DAI was calculated, taking into account the mean value of the following scores: body weight score (0 to 4: no weight loss, 1%–5% weight loss; 5%–10% weight loss; 10%–20% weight loss, and over 20% weight loss), stool consistency score (0 to 4: normal stool, mild loose stool, loose stool, mild diarrhea, and diarrhea). and occult blood score (0 to 4: no blood, mild bleeding, moderate bleeding, severe bleeding, and gross bleeding). This scoring system aligns with the methodology outlined in a previous study.^26^

### 2.3 Hematoxylin and eosin (HE) staining

Pathological changes were evaluated in colon sections prepared from paraffin‐embedded samples using the HE staining kit (C0105S; Beyotime Biotechnology Co., Ltd.). In short, the sections were deparaffined, rehydrated, and stained with hematoxylin for 5 min, followed by eosin staining for 2 min. The stained sections were dehydrated, cleared in xylene, and sealed with neutral balsam for microscopic observation.

DSS‐induced colon injury was evaluated by two pathologists unaware of the grouping details utilizing a histological scoring system. The system comprised three independent parameters including inflammation severity (0 to 3: none, mild, moderate, and severe inflammatory cell infiltration, respectively), injury depth (0 to 3: none, injury restricted to the mucosal layer, injury in mucosal and submucosal layers, and transmural injury), and crypt‐epithelial damage (0 to 4: none, basal one‐third damaged, basal two‐thirds damaged, only surface epithelium intact, and entire crypt and epithelium lost). The sum of the three independent scores constituted the final histology score.^27^

### 2.4 Immunohistochemistry (IHC)

Paraffin‐embedded colon tissue sections (5 μm) were dewaxed, rehydrated, treated with 3% H_2_O_2_, heat‐bathed in citrate buffer for antigen retrieval, and blocked with normal goat serum. Subsequently, the sections were incubated with antibodies of mucin 3 (MUC3; 1:500, PAB031Mu01; Cloud‐Clone Corp.), Claudin‐1 (1:500, ab211737; Abcam Inc.), and tight junction protein 1 (ZO‐1, 1:500, ab276131; Abcam) overnight at 4°C, followed by incubation with goat anti‐rabbit IgG (1: 1,000, ab6721; Abcam) at 37°C for 20 min. The sections were then treated with horseradish peroxidase (HRP)‐labeled streptavidin‐working solution (A0303; Beyotime) and developed by DAB (P0202; Beyotime). Following counter‐staining with hematoxylin, the sections were observed under a microscope to calculate the number of positive cells in five randomly selected fields.

### 2.5 Cell culture and in‐vitro inflammation models

The human colonic epithelial cell line NCM‐460 (BNCC339288) was procured from BeNa Culture Collection. The cells were cultured in Dulbecco's modified Eagle's medium (DMEM; Cat. No. 11965092) containing 1% antibiotics (Cat. No. 15140122) and 10% fetal bovine serum (10099141C) (all provided by Thermo Fisher Scientific) at 37°C with 5% CO_2_. The cells were passaged when the confluence reached 80%–90%.

Nexrutine was used to treat NCM‐460 cells at 10 μg/mL according to previous reports,^28^, ^29^ and another concentration of 20 μg/mL was set up to obtain more comprehensive results. Mesalazine was administered at a concentration of 40 mM to suppress inflammatory responses.^30^

Lipopolysaccharide (LPS; L23352, Thermo Fisher Scientific) was used to induce inflammation in cells. The NCM‐460 cells were allocated into the following groups: Normal group, LPS group (200 ng/mL LPS treatment for 24 h), LPS + 10 µg/mL Nexrutine group (200 ng/mL LPS and 10 µg/mL treatment for 24 h), LPS + 20 µg/mL Nexrutine group (200 ng/mL LPS and 20 µg/mL Nexrutine treatment for 24 h), LPS + Mesalazine group (200 ng/mL LPS and 40 mM Mesalazine treatment for 24 h), LPS + Nexrutine + oe‐NC group (pretransfection of oe‐NC, followed by 200 ng/mL LPS and 20 µg/mL Nexrutine treatment for 24 h), and LPS + Nexrutine + oe‐RELA group (pretransfection of oe‐NC, followed by 200 ng/mL LPS and 20 µg/mL Nexrutine treatment for 24 h). The oe‐NC and oe‐RELA plasmids were constructed by Sangon Biotech Co., Ltd. according to the known RELA sequence (NM_001145138.2) in NCBI, which were transfected into the NCM‐460 cells according to the instructions of Lipofectamine™ 2000 (Cat. No. 11668027; Thermo Fisher Scientific). The cells were harvested for subsequent treatments 48 h after transfection.

### 2.6 Enzyme‐linked immunosorbent assay (ELISA)

Concentrations of inflammatory cytokines (TNF‐α, IL‐1β, and IL‐6) and anti‐inflammatory cytokine IL‐10 in the homogenate of mouse clone tissue were analyzed using the mouse TNF‐α (ab108910; Abcam), mouse IL‐1β (ab197742; Abcam), mouse IL‐6 (ab100713; Abcam), and mouse IL‐10 (EM0100; Wuhan Fine Biotech Co., Ltd.) ELISA kits. The concentrations of TNF‐α, IL‐1β, and IL‐6 in the culture supernatant of NCM‐460 cells were analyzed by human TNF‐α (ab181421; Abcam), human IL‐1β (ab214025; Abcam), and human IL‐6 (ab178013; Abcam) ELISA kits. The optical density (OD) value at 450 nm read by a Multiskan SkyHigh full‐wavelength microplate reader (A51119700DPC; Thermo Fisher Scientific).

### 2.7 Cell counting kit‐8 (CCK‐8) method

The NCM‐460 cells were seeded in 96‐well plates at 10^4^ cells per well for 24 h. Subsequently, each well was loaded with 10 μL CCK‐8 reagent (C0038; Beyotime), followed by an additional 1‐h incubation in a humidified incubator (37°C). The OD value at 450 nm was read by the microplate reader. Relative cell viability was determined by normalizing the OD values to the OD value of the control group.

### 2.8 Immunofluorescence staining

The NCM‐460 cells were fixed in 4% paraformaldehyde for 10 min, penetrated with 0.2% Triton X‐100 for 10 min, quenched by 100 mM for 10 min, and blocked by 5% bovine serum albumin (BSA). Subsequently, the cells were incubated with antibodies of Claudin‐1 (1:1000, ab211737; Abcam) and ZO‐1 (1:100, ab276131; Abcam), and then with Alexa Fluor 594‐conjugated antibody (1:100, ab150080; Abcam). The nuclei were counter‐stained with DAPI (C1002; Beyotime) for 10 min, and then the staining was observed under the EVOS M5000 fluorescence microscope (AMF5000; Thermo Fisher Scientific).

### 2.9 RNA isolation and quantification

Total RNA from mouse colon tissues or NCM‐460 cells was isolated using TRIzol (Cat. No. 16096020; Thermo Fisher Scientific), and reverse transcription was performed using the PrimeScript RT Reagent Kit (RR047A; Takara Holdings Inc.). Subsequently, quantitative polymerase chain reaction (qPCR) was conducted using the TB green® Premix Ex Taq™ II kit (RR820A, Takara). All procedures strictly followed the manufacturer's instruction manuals. Gene expression relative to the endogenous control (GAPDH) was evaluated by the $2^{-∆∆C_{t}}$ method. The primers used were below: RELA (mouse): F: 5ʹ‐CCTCGGGACAAACAGCCTC‐3ʹ, R: 5ʹ‐CACGGCGCGCTAAAGTAAAG‐3ʹ; RELA (human): F: 5ʹ‐CCCTTCCAAGAAGAGCAGCG‐3ʹ, R: 5ʹ‐TCACTCGGCAGATCTTGAGC‐3ʹ; GAPDH (mouse): F: 5ʹ‐CCCTTAAGAGGGATGCTGCC‐3ʹ, R: 5ʹ‐TACGGCCAAATCCGTTCACA‐3ʹ; GAPDH (human): F: 5ʹ‐AATGGGCAGCCGTTAGGAAA‐3ʹ, R: 5ʹ‐GCGCCCAATACGACCAAATC‐3ʹ.

### 2.10 Western blot (WB) analysis

Mouse colon tissues or NCM‐460 cells were lysed using the radio‐immunoprecipitation assay lysis kit (R0010; Solarbio Science & Technology Co., Ltd.) to isolate total protein. Protein concentration was determined using the bicinchoninic acid kit (AR1189; Boster Biological Technology Co., Ltd.). The protein samples were separated by 10% SDS‐PAGE and transferred onto polyvinylidene fluoride membranes. After blocking with 5% BSA for 2 h, the membranes were probed with diluted primary antibodies against RELA (1:1000, 80979‐1‐RR; Proteintech Group, Inc.) and GAPDH (1:1,000, ab9485; Abcam) at 4°C overnight, followed by incubation with HRP‐labeled secondary antibody (1:1,000, ab6721; Abcam) at room temperature for 1 h. The protein bands were developed using the Pierce™ ECL kit (Cat. No. 32209; Thermo Fisher Scientific), and protein expression, relative to GAPDH, was analyzed by Image J software.

### 2.11 Statistical analysis

All statistics were performed by Prism 8.0.2 (GraphPad). Measurement data are presented as the mean ± standard deviation. Intergroup differences were assessed through the unpaired *t* test, or through one‐ or two‐way analysis of variance (ANOVA), followed by Tukey's post‐hoc examination in the case of multiple groups. A significance level of *p* < .05 was considered statistically significant.

## 3 RESULTS

### 3.1 Nexrutine alleviates inflammation and colonic mucosal barrier damage in mice with UC

**Figure 1:**
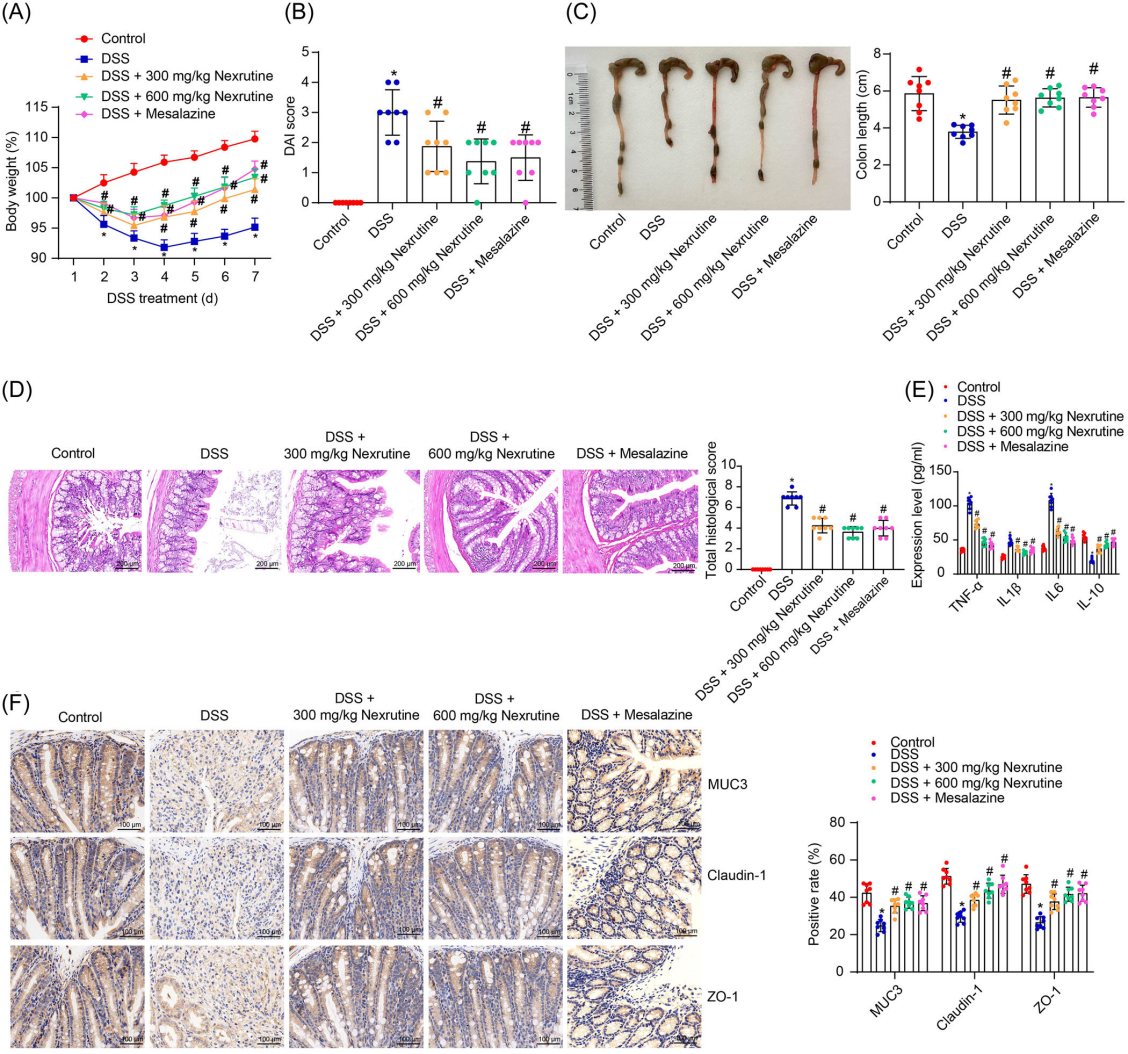
Nexrutine alleviates inflammation and colonic mucosal barrier damage in mice with UC. A mouse model of UC was induced by administration of 3% DSS in drinking water. The model mice were treated with Nexrutine (300 mg/kg or 600 mg/kg) or the positive control regimen Mesalazine (195 mg/kg). (A) Body weight change of mice in each group; (B) DAI score of mice in each group; (C) length of whole colon of mice in each group; (D) pathological injury score in mouse colon tissues evaluated by HE staining; (E) concentrations of IL‐1β, IL‐6, TNF‐α, and IL‐10 in mouse colon tissues analyzed by ELISA kits; (F) expression of MUC3, Claudin‐1, and ZO‐1 in mouse colon tissues determined by IHC assay. In each group, *n* = 8. Differences were analyzed by the one‐way (B, C, D) or two‐way (A, E, F) ANOVA. *p < .05 versus the control group; ^#^ *p* < .05 versus the DSS group. DAI, disease activity index; DSS, dextran sulfate sodium; HE, hematoxylin and eosin; IHC, immunohistochemistry; UC, ulcerative colitis.

A mouse model of UC was induced by administering of 3% DSS in drinking water. Compared with the control mice, the DSS‐induced mice exhibited significant weight loss, along with varying degrees of diarrhea and blood in the stool, corresponding to increased DAI scores (*p* < .0001) (Figure 1A,B). Treatment with Nexrutine or the positive control regimen Mesalazine (195 mg/kg) significantly restored the body weight of the DSS‐challenged mice (all *p* < .0001) and lowered the DAI scores (300 mg/kg Nexrutine: *p* = .0007; 600 mg/kg Nexrutine: *p* < .0001; Mesalazine: *p* < .0001). (Figure 1A,B). After euthanasia, the whole colon tissues of mice were collected. It was found that the length of colons was significantly reduced by DSS treatment (*p* < .0001), a phenomenon ameliorated by Nexrutine or Mesalazine treatment (all *p* < .0001) (Figure 1C). HE staining showed that DSS induction led to a substantial thickening of the colonic wall, pronounced inflammatory cell infiltration, loss of crypt fossa, impairment of mucosal barrier, tissue edema, and a notable increase in the overall tissue damage score (*p* < .0001). These pathological changes were largely mitigated by Nexrutine or Mesalazine treatment (all *p* < .0001) (Figure 1D). The ELISA results showed that the concentrations of pro‐inflammatory cytokines including IL‐1β, IL‐6, and TNF‐α in mouse colon tissues were increased after DSS treatment, accompanied by a significant decrease in IL‐10 (all *p* < .0001). Treatment of Nexrutine at different doses or Mesalazine significantly reduced the concentrations of IL‐1β (300 mg/kg Nexrutine: *p* = .0006; 600 mg/kg Nexrutine: *p* < .0001; Mesalazine: *p* = .0047), IL‐6 (all *p* < .0001), and TNF‐α (all *p* < .0001) while restoring the concentration of IL‐10 (all *p* < .0001) (Figure 1E). Moreover, IHC assay was performed to analyze the expression of MUC3, barrier‐related transmembrane protein Claudin‐1, and tight junction protein ZO‐1 in the mouse colon tissues. DSS‐treated mice exhibited significantly decreased staining of MUC3, Claudin‐1, and ZO‐1 (all *p* < .0001). Treatment with Nexrutine at different doses or Mesalazine significantly increased the positive staining of MUC3 (all *p* < .0001), Clausin‐1 (300 mg/kg Nexrutine: *p* = .0001; 600 mg/kg Nexrutine: *p* < .0001; Mesalazine: *p* < .0001), and ZO‐1 (all *p* < .0001) (Figure 1F).

### 3.2 Nexrutine reduces LPS‐induced damage in NCM‐460 cells

**Figure 2:**
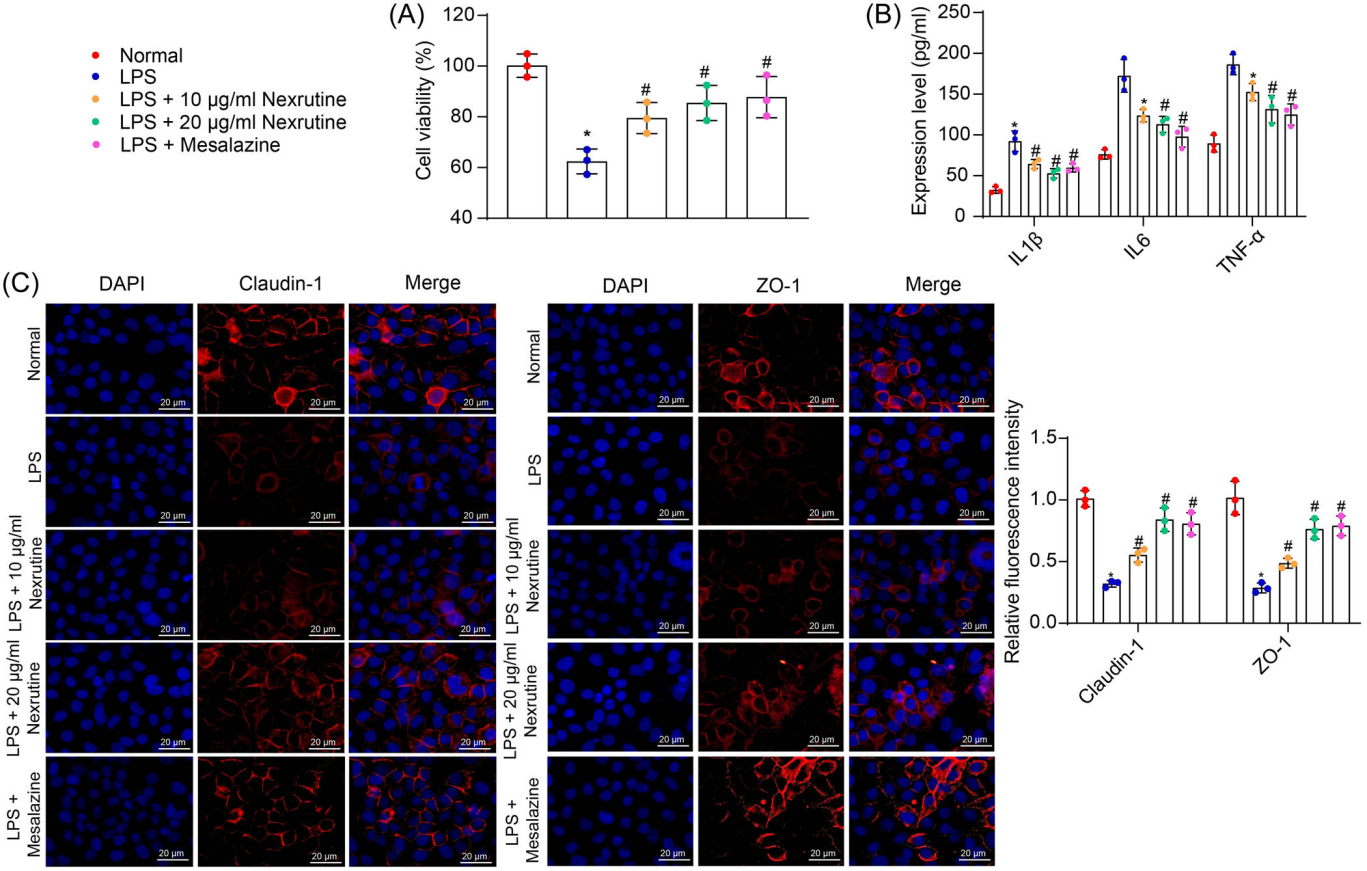
Nexrutine reduces LPS‐induced damage in NCM‐460 cells. NCM‐460 cells were treated with LPS (200 ng/mL) to simulate an inflammatory condition, alongside with treatments of Nexrutine (10 and 20 μg/mL) or Mesalazine (40 mM). (A) Viability of NCM‐460 cells determined by CCK‐8 assay; (B) concentrations of IL‐1β, IL‐6, and TNF‐α in the culture supernatant of NCM‐460 cells analyzed by ELISA; (C) expression of Claudin‐1 and ZO‐1 in the NCM‐460 cells determined by immunofluorescence staining. Three biological replicates were performed. Differences were compared by the one‐way (A) or two‐way (B‐C) ANOVA. *p < .05 versus the Normal group; ^#^ *p* < .05 versus the LPS group.

In vitro, NCM‐460 cells were treated with LPS to simulate an inflammatory condition. Concurrently, Nexrutine was administered at concentrations of 10 and 20 μg/mL, while Mesalazine was used at 40 mM as the positive control. The CCK‐8 results revealed a significant reduction in cell viability upon LPS stimulation (*p* = .0002), which was restored by 10 μg/mL Nexrutine (*p* = .0462), 20 μg/mL Nexrutine (*p* = .0079), or Mesalazine (*p* = .0041) (Figure 2A). Consistent with the in vivo observations, ELISA showed that concentrations of IL‐1β, IL‐6, and TNF‐α were conspicuously elevated in the culture supernatant of LPS‐treated NCM‐460 cells (all *p* < .05). Treatment with Nexrutine or Mesalazine significantly reduced the concentrations of IL‐1β (10 μg/mL Nexrutine: *p* = .0328; 20 μg/mL Nexrutine: *p* = .013; Mesalazine: *p* = .0092), IL‐6 (all *p* < .0001), and TNF‐α (10 μg/mL Nexrutine: *p* = .0067; 20 μg/mL Nexrutine: *p* < .0001; Mesalazine: *p* < .0001) (Figure 2B). Additionally, immunofluorescence staining showed that the expression of Claudin‐1 and ZO‐1 was significantly decreased in the LPS‐stimulated NCM‐460 cells (all *p* < .0001). T Treatment with Nexrutine or Mesalazine once again significantly restored the staining intensity of Claudin‐1 (10 μg/mL Nexrutine: *p* = .0154; 20 μg/mL Nexrutine: *p* < .0001; Mesalazine: *p* < .0001) and ZO‐1 (10 μg/mL Nexrutine: *p* = .0414; 20 μg/mL Nexrutine: *p* < .0001; Mesalazine: *p* < .0001) (Figure 2C).

### 3.3 Nexrutine suppresses RELA transcription

**Figure 3:**
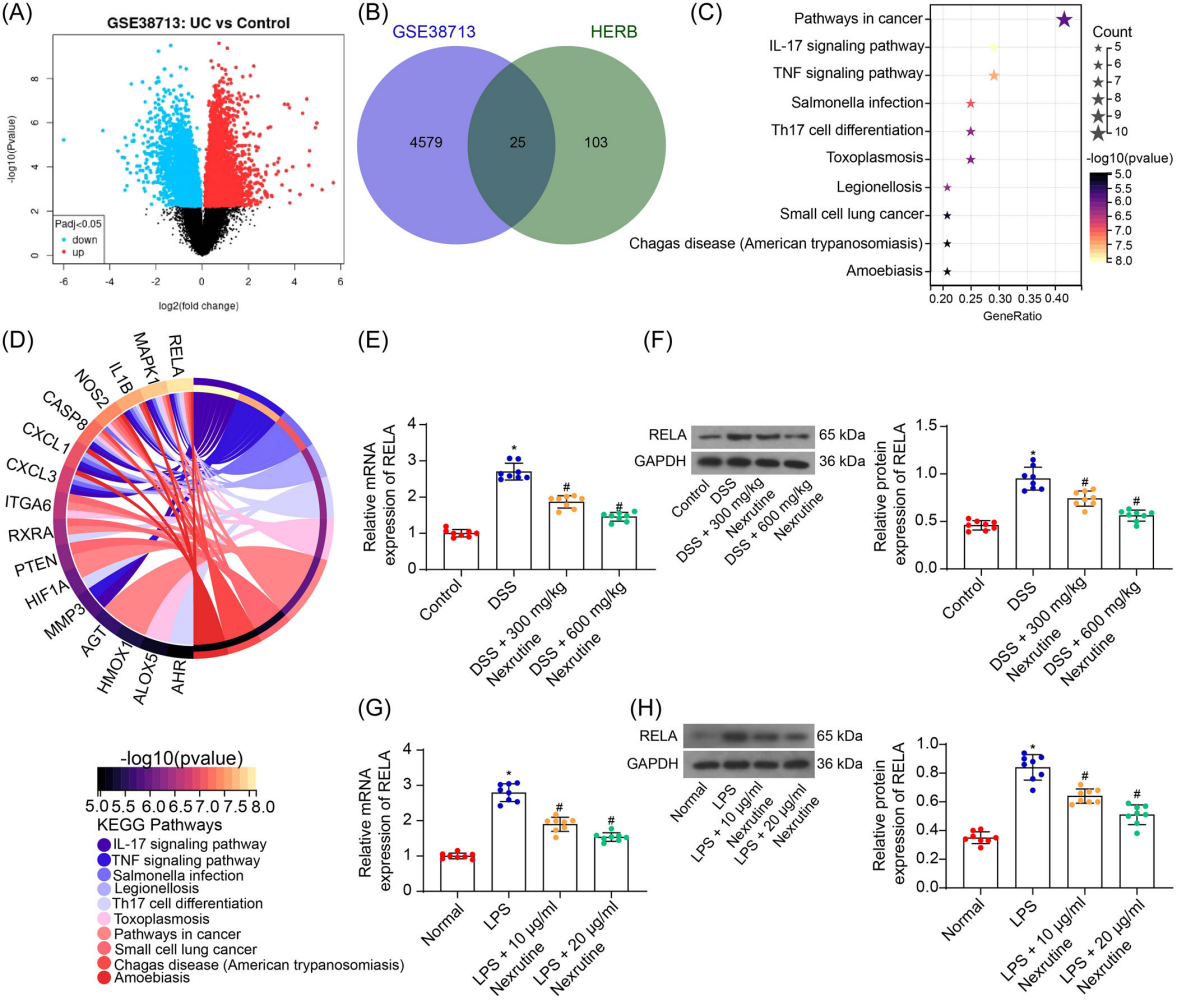
Nexrutine suppresses RELA transcription. (A) DEGs between diseased colon tissues and control tissues screened from the GSE38713 data set; (B) intersections of the possible gene targets of Nexrutine and the DEGs in (A); (C, D) KEGG pathway enrichment analysis of the 25 intersecting genes; (E, F) mRNA (E) and protein (F) levels of RELA in mouse colon tissues determined by qPCR and WB assays; (G, H) mRNA (G) and protein (H) levels of RELA in NCM‐460 cells determined by qPCR and WB assays. For animal assays, *n* = 8 in each group; for cellular assays, three biological replicates were performed. Differences were compared by the one‐way (E–H) ANOVA. *p < .05 versus the Control or Normal group; ^#^ *p* < .05 versus the DSS or LPS group. DEG, differentially expressed genes.

To explore the functional targets modulated by Nexrutine in alleviating UC, we analyzed transcriptome changes in UC using the GEO GSE38713 data set (UC: GSM948563~GSM948592; Control: GSM948550~GSM948562). Differentially expressed genes (DEGs) between diseased colon tissues and control tissues were screened with a significance threshold of *p* < .05 (Figure 3A). The DEGs were cross‐referenced with the gene targets of Nexrutine predicted in the HERB database (http://herb.ac.cn/), revealing 25 candidate genes at the intersection (Figure 3B). Kyoto Encyclopedia of Genes and Genomes (KEGG) enrichment analysis on these genes highlighted two signaling pathways with high confidence levels: ko04657: IL‐17 signaling pathway and ko04668: TNF signaling pathway. Intriguingly, the genes enriched in both pathways were completely the same: MMP3, RELA, CASP8, CXCL3, CXCL1, IL1B, and MAPK (Figure 3C,D), with RELA (p65) plays a central regulatory role in both pathways (Supporting Information: Figure S1A,B). Consequently, we hypothesized that RELA is one of the core targets affected by Nexrutine during inflammation resolution and UC alleviation.

Compared with the control mice, DSS‐treated mice exhibited significantly elevated mRNA and protein levels of RELA in their colon tissues (all *p* < .0001), which were reduced by Nexrutine treatments at both doses (all *p* < .0001) (Figure 3E,F). Similar results were observed in vitro, where mRNA and protein levels of RELA were increased in NCM‐460 cells after LPS treatment (all *p* < .0001) but were then decreased by 10 or 20 μg/mL of Nexrutine treatment (all *p* < .0001) (Figure 3G,H).

### 3.4 Overexpression of RELA exacerbates LPS‐induced inflammation in Nexrutine‐treated NCM‐460 cells

**Figure 4:**
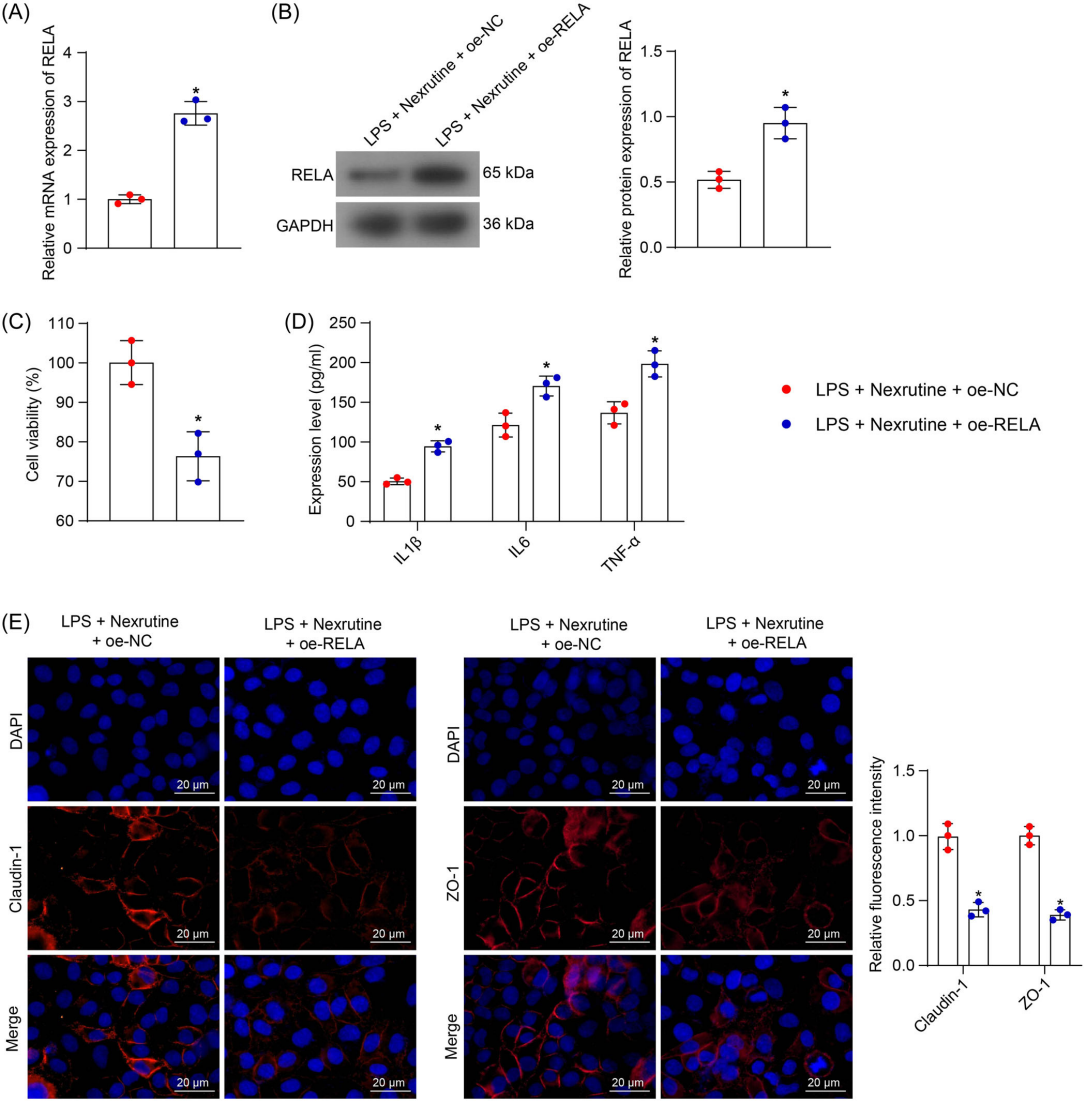
Overexpression of RELA aggravates LPS‐induced inflammation in Nexrutine‐treated NCM‐460 cells. NCM‐460 cells were transfected with oe‐RELA, followed by LPS (200 ng/mL) and Nexrutine (20 ng/mL) treatments. (A, B) mRNA (A) and protein (B) levels of RELA in NCM‐460 cells determined by qPCR and WB assays; (C) viability of NCM‐460 cells analyzed by CCK‐8 method; (D) concentrations of IL‐1β, IL‐6, and TNF‐α in the culture supernatant of NCM‐460 cells determined by ELISA kits; (E) expression of Claudin‐1 and ZO‐1 in the NCM‐460 cells determined by immunofluorescence staining. Three biological replicates were performed. Differences were compared by the unpaired *t* test (A, B, C) or two‐way ANOVA (D, E). *p < .05 versus the LPS + Nexrutine + oe‐NC group.

To validate whether Nexrutine suppresses RELA to relieve UC‐related symptoms, we induced RELA overexpression in NCM‐460 cells. The successful upregulation of RELA mRNA (*p* = .0003) and protein (*p* < .0001) was confirmed by the qPCR and WB assays (Figure 4A,B). These cells were then treated with LPS and 20 μg/mL Nexrutine. Compared with oe‐NC, the viability of cells was significantly reduced by oe‐RELA (Figure 4C). The RELA overexpression also increased the concentrations of IL1β (*p* = .0027), IL6 (*p* = .0011), and TNF‐α (*p* = .0002) in the culture supernatant (Figure 4D) and reduced the intensities of immunofluorescence staining of Claudin‐1 (*p* < .0001) and ZO‐1 (*p* < .0001) in the NCM‐460 cells (Figure 4E). In essence, the anti‐inflammatory and protective properties of Nexrutine on NCM‐460 cells were counteracted upon RELA upregulation.

### 3.5 Restoration of RELA exacerbates inflammatory injury in mouse colon tissues

**Figure 5:**
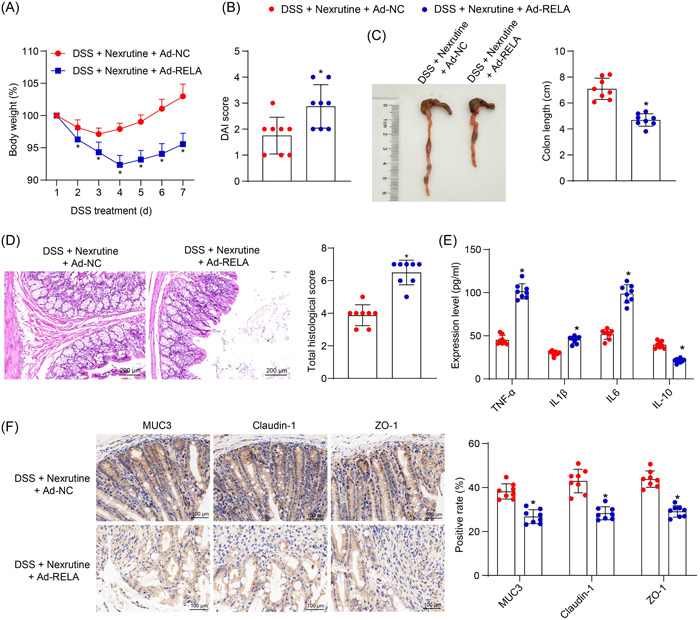
Restoration of RELA aggravates inflammatory injury in mouse colon tissues. Mice were injected with Ad‐RELA through the mesenteric artery, followed by the 3% DSS and Nexrutine (300 mg/kg) treatments. (A) Body weight change of mice in each group; (B) DAI score of mice in each group; (C) length of whole colon of mice in each group; (D) pathological injury score in mouse colon tissues evaluated by HE staining; (E) concentrations of IL‐1β, IL‐6, TNF‐α, and IL‐10 in mouse colon tissues analyzed by ELISA kits; (F) expression of MUC3, Claudin‐1, and ZO‐1 in mouse colon tissues determined by IHC assay. In each group, *n* = 8. Differences were analyzed by the unpaired *t* test (B–D) or two‐way ANOVA (A, E, F). *p < .05 versus the control group; ^#^ *p* < .05 versus the DSS group. DAI, disease activity index; DSS, dextran sulfate sodium; HE, hematoxylin and eosin; IHC, immunohistochemistry.

In vivo, overexpression of RELA was induced by mesenteric artery injection of Ad‐RELA, followed by the 3% DSS and Nexrutine treatments. Compared with Ad‐NC, the additional administration of Ad‐RELA led to a significant body weight loss (*p* < .0001, diarrhea, and blood in the stool, namely increased DAI scores (*p* < .0001) (Figure 5A,B). Moreover, the Ad‐RELA‐treated mice showed shortened colons and deteriorated mucosal tissue damage (all *p* < .0001) (Figure 5C,D). Moreover, the Ad‐RELA administration significantly enhanced the concentrations of IL1β, IL6, and TNF‐α while decreasing the concentration of IL‐10 in the colon tissue homogenate of mice (Figure 5E). At the meantime, the IHC assay on the colon tissues showed that the Ad‐RELA led to a conspicuous downregulation of the MUC3, Claudin‐1, and ZO‐1 proteins (*p* < .0001) (Figure 5F). Therefore, it can be inferred that the treating effect of Nexrutine on UC is due in part to the downregulation of RELA.

## 4 DISCUSSION

Patients with inflammatory bowel disease are reportedly to be increasingly turning to herbal products as complementary or alternative medicines.^31^ The experiments in this study reveal that Nexrutine demonstrated beneficia effects in resolving inflammatory responses and maintaining mucosal barrier integrity in mice with UC and in LPS‐irritated human NCM‐460 cells, with the involvement of RELA suppression.

Traditional herbal medicines have been demonstrated as promising complementary or alternative therapeutic regimens for UC.^32^, ^33^ Various herbal or plant extracts have exhibited therapeutic effects on UC in several randomized, double‐blind clinical studies. For instance, treatment with wheat grass juice (*Triticum aestivum*) resulted in endoscopic improvement in the majority of patients with active UC, accompanied by reduced DAI and alleviated rectal bleeding.^34^ Oral administration of *Aloe vera* reduced the histological disease activity in patients with mild to moderate UC compared with those treated with a placebo.^35^ Other herbs, such as *Andrographis paniculate* and *Boswellia serrata*, exhibited similar protective functions in clinical trials.^36^, ^37^ Shenling BaiZhu powder, a formula composed of various herbal medicines like *Atractylodes macrocephala* Koidz., *Panax ginseng* C.A. Mey., *Glycyrrhiza uralensis* Fisch., *Amomum villosum* Lour., *Poria cocos* Wolf., *Platycodon grandiflorum* A. DC., *Dioscorea opposita* Thunb., *Nelumbo nucifera* Gaertn., and *Dolichos lablab* L., has been recently suggested as a promising regimen in the prevention and treatment of UC.^38^ As previously mentioned, Nexrutine is reported to play a relieving role in inflammation, abdominal pain, gastroenteritis, and diarrhea. For safety concerns, a prior test showed that the administration of Nexrutine at moderate doses in rats (250, 500, 750 mg/kg) for continuous 28 days did not result in any sign of toxicity.^39^ In this study, we treated the 3% DSS‐induced C57BL/6 J mice with Nexrutine at doses of 300 and 600 mg/kg, respectively. Both doses reduced weight loss, diarrhea, and blood in the stool of mice, alleviating histological pathological changes in the mouse colon tissues. Meanwhile, Nexrutine treatments were found to reduce pro‐inflammatory cytokines and restore mucosal barrier integrity‐related factors both in the mouse colon tissues and in LPS‐induced NCM‐460 cells. These results are consistent with a previous report by Choi et al., demonstrating that Nexrutine suppressed inflammatory cytokines (TNF‐α, IL‐6, and IL‐8) and rescued cell adhesion components (ZO‐1 and Occludin) in PM2.5 particulate matter‐exposed human keratinocytes by suppressing proteinase‐activated receptor‐2 generation.^40^ The current evidence validates the significant therapeutic efficacy of Nexrutine in mouse and human cell models of UC.

Through cross‐screening using several bioinformatics tools, RELA was identified as a promising target gene of Nexrutine, and a significant downregulation of RELA mRNA and protein was observed in both Nexrutine‐treated mice and NCM‐460 cells. Indeed, Nexrutine has been found to suppress the expression and phosphorylation of RELA in gastric cancer cells, leading to a less malignant phenotype.^16^ Similarly, a previous study by Choi et al. claimed that the Nexrutine exerts anti‐inflammatory properties by inactivating the NF‐κB and mitogen‐activated protein kinase signaling pathways.^41^ Mounting evidence has demonstrated the beneficial effects of RELA (p65) on the intestinal epithelial cell survival, inflammation resolution, and intestinal barrier maintenance in UC.^42^, ^43^, ^44^ In this study, we identified that the artificial upregulation of RELA, both in the 3% DSS‐induced mice and LPS‐induced NCM‐460 cells, significantly blocked the alleviating roles of Nexrutine from all aspects.

Nevertheless, there are several shortcomings that should be acknowledged. We used two independent doses of Nexrutine for both animal and cellular experiments. This setup may be considered limited for drawing a conclusive determination of Nexrutine's dose‐dependent effects on tissue/cell protection or establishing the optimal clinical dose. Furthermore, this study focuses on RELA as a target of Nexrutine. There might be more core factors involved in the Nexrutine‐mediated events, which should be further investigated. Additionally, the present study primarily focuses on the ameliorating effects of Nexrutine against self‐inflammatory damage of colonic epithelial cells. Considering the observations that Nexrutine reduced T helper (Th) 1 cell cytokines (TNF‐α, IL‐1β and IL‐6) while elevating the Th2 cell cytokine (IL‐10) in mouse clone tissues, and the NF‐κB activation is closely correlated with lymphocytes and other inflammatory cells,^45^ Nexrutine may exert anti‐inflammatory functions through other pathways. However, this issue was not further concerned in the present study and is interesting for additional investigations. We intend to focus on these issues in our future research endeavors to provide a more comprehensive understanding of the efficacy of Nexrutine in UC management.

## 5 CONCLUSION

**Figure 6:**
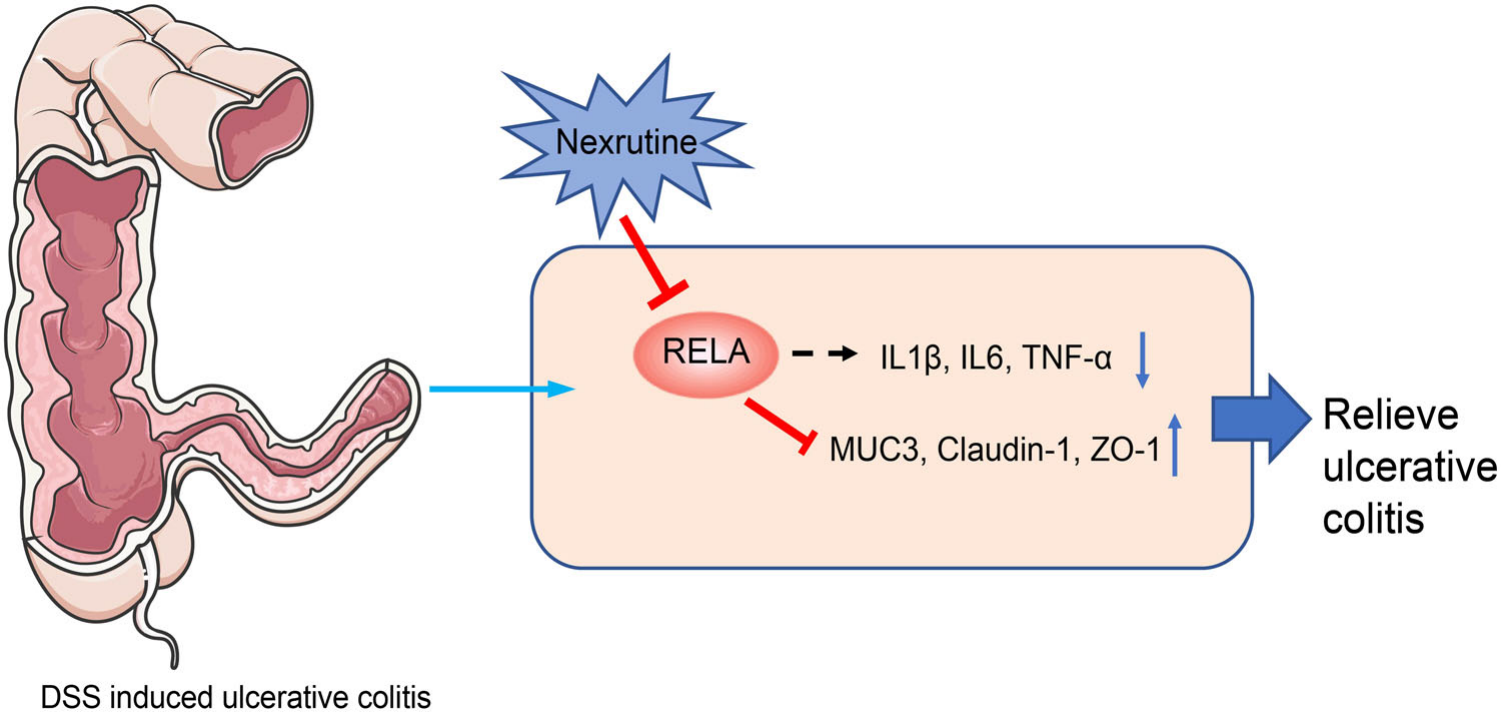
Nexrutine suppresses RELA transcription, therefore reducing the production of pro‐inflammatory cytokines, improving the colonic barrier, and alleviating UC‐like symptoms. UC, ulcerative colitis.

In conclusion, this preclinical research highlights the promising therapeutic potential of Nexrutine in the management of inflammatory mucosal injury in UC. The involvement of RELA suppression, identified through cross‐screening using bioinformatics tools, sheds light on the molecular mechanisms underlying Nexrutine's beneficial effects (Figure 6). Nexrutine's performance in mitigating UC symptoms aligns with previous reports on herbal extracts, emphasizing its role as a valuable addition to complementary or alternative therapies for UC. The study contributes to the growing body of evidence supporting herbal interventions in the management of inflammatory diseases. However, dose‐dependent effects and optimal clinical dosage warrant further exploration. Future research should address the remaining gaps to provide a more comprehensive understanding of Nexrutine's efficacy in UC management and pave the way for its clinical translation as a promising therapeutic option.

## AUTHOR CONTRIBUTIONS

Hongyun Xu conceptualized and designed the study and administered the long study project and acquired data. Chunyu Wu and Danning Wang analyzed the data and wrote the original draft. Haiqiang Wang reviewed and interpreted the results and provided editing. All authors read and approved the final manuscript.

## CONFLICT OF INTEREST STATEMENT

The authors declare no conflict of interest.

## Supporting information

Supporting information.

## Data Availability

The data set generated and/or analyzed during the current study are available from the corresponding author on reasonable request.
