# Supplementary material for: Alleviating effect of Nexrutine on mucosal inflammation in mice with ulcerative colitis: Involvement of the RELA suppression
Source: Immun Inflamm Dis. 2024 Jan 19;12(1):e1147. doi: 10.1002/iid3.1147 (PMC10797652; doi:10.1002/iid3.1147)
Supplement: Supplementary file 1 — Supporting information. [file IID3-12-e1147-s001.docx]

**Figure S1**

**
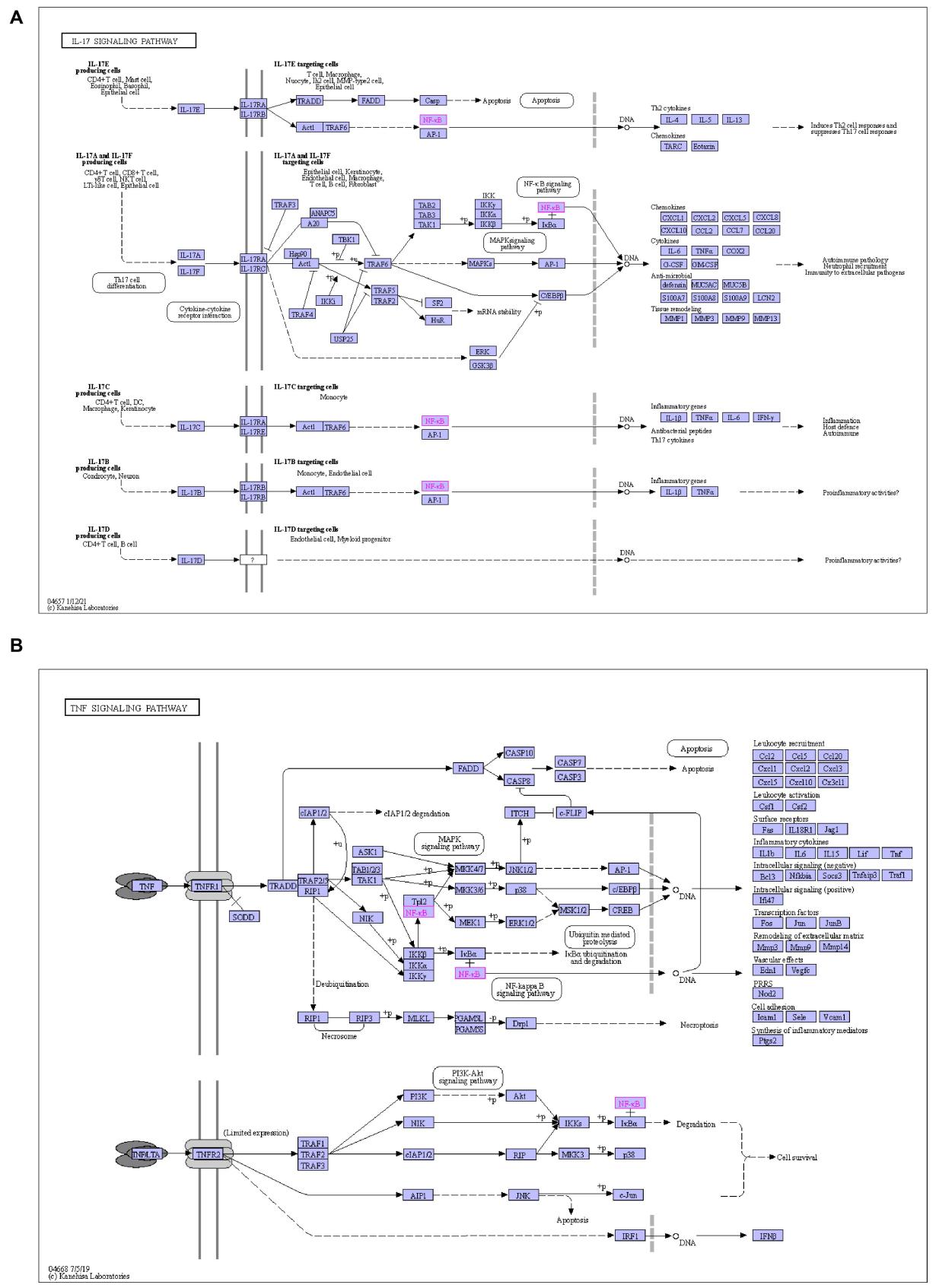
**

**Fig S1** Signaling transduction of the significantly enriched pathways. A, signaling transduction diagram of the ko04657: IL-17 signaling pathway; B, signaling transduction diagram of the ko04668：TNF signaling pathway.
